# Shared Decision-Making. A Primer for Clinicians

**DOI:** 10.1007/s11606-025-09707-z

**Published:** 2025-10-07

**Authors:** Glyn Elwyn, Pål Gulbrandsen, Hannah Leavitt, Eman Abukmail, Marla L. Clayman, Adrian Edwards, Jeanette Finderup, Alana Fisher, Stuart W. Grande, Pola Hahlweg, Tammy Hoffmann, Wen-Hsuan Hou, María José Hernández-Leal, Debra Leung, Weiwei Lu, Lars Mandelkow, Kristen E. Pecanac, Arwen H. Pieterse, Amy Price, Jannicke Rabben, Paula Riganti, Michael Sanatani, Fülöp Scheibler, Elise Schoefs, Owen A. Taylor, Kathrene D. Valentine, Richard Wexler

**Affiliations:** 1 The Dartmouth Institute for Health Policy & Clinical Practice, Dartmouth College, WTRB, Lebanon, NH USA; 2 Institute of Clinical Medicine, University of Oslo, HØKH Health Services Research Unit, Akershus University Hospital, and Senior Researcher, Nordbyhagen, Norway; 3 Institute for Evidence-Based Healthcare, Bond University, Gold Coast, QLD Australia; 4 Center for Health Organization and Implementation Research (CHOIR), Veterans Health Administration, Washington, DC USA; 5 Division of Population Medicine, Cardiff University, Neuadd Meirionnydd, Cardiff, UK; 6 Department of Medicine and Nephrology, Aarhus University, Aarhus, Denmark; 7 eCentreClinic, School of Psychological Sciences, Macquarie University, Sydney, Australia; 8 Division of Health Policy and Management, School of Public Health, University of Minnesota, Minneapolis, USA; 9 Department of Medical Psychology, University Medical Center Hamburg-Eppendorf, Hamburg, Germany; 10 Institute for Evidence-Based Healthcare, Bond University, Gold Coast, QLD Australia; 11 Department of Physical Medicine and Rehabilitation, Taipei Medical University Hospital, Taipei City, Taiwan; 12 Department of Community, Maternity and Pediatric Nursing, School of Nursing, University of Navarra, Pamplona, Spain; 13 Department of Anaesthesia, Perioperative Medicine and Pain Medicine. Peter MacCallum Cancer Centre, Melbourne, VIC Australia; 14 Department of Social and Behavioural Sciences, City University of Hong Kong, Kowloon Tong, HKSAR China; 15 Centre for Shared Decision Making, University Hospital of North Norway, Tromsø, Norway; 16 School of Nursing, University of Wisconsin-Madison, Madison, WI USA; 17 Medical Decision Making, Department of Biomedical Data Sciences, Leiden University Medical Center, Leiden, Netherlands; 18 Faculty of Medicine, University of Oslo, Oslo, Norway; 19 Dartmouth Health, Lebanon, NH USA; 20 Faculty of Health and Sport Science, Department of Health and Nursing Science, University of Agder, Kristiansand, Norway; 21 Family and Community Medicine Department Hospital Italiano de Buenos Aires, Perón, Buenos Aires, Argentina; 22 Division of Medical Oncology, Schulich School of Medicine & Dentistry, Western University, London, ON Canada; 23 National Competency Center for Shared Decision Making, University Medical Center Schleswig-Holstein, Kiel, Germany; 24 Department of Pharmaceutical and Pharmacological Sciences, KU Leuven, Leuven, Belgium; 25 Cardiovascular Epidemiology Unit, Department of Public Health and Primary Care, Victor Phillip Dahdaleh Heart and Lung Research Institute, Cambridge, UK; 26 Department of General Internal Medicine, Massachusetts General Hospital, Boston, MA USA; 27 Informed Medical Decisions Foundation, Boston, MA USA

**Keywords:** shared decision-making, patient-centered care, co-production

## Abstract

**Importance:**

Shared decision-making is a widely promoted approach, yet clinicians, typically supportive in principle, find it difficult to implement because of concerns and barriers they commonly encounter in practice.

**Objective:**

To generate a primer that describes shared decision-making from the perspective of clinicians.

**Methods:**

We collaborated with clinicians, patient representatives, and health service researchers. We invited members of the International Society of Shared Decision Making to co-produce a primer for clinicians using a series of jointly edited online documents. We shared drafts with other clinicians and patients. Finally, we integrated the contributions until we had arrived at a consensus.

**Findings:**

Twenty-five people from 13 countries contributed; 9 had medical qualifications, 4 had nursing qualifications, and 12 others had a range of backgrounds. A total of 30 patients and clinicians provided further comments. The description differs from previous versions because it addresses the *barriers* that clinicians frequently mention. It describes how to overcome common challenges by emphasizing the importance of a clear invitation at initiation; it suggests how to manage patients’ resistance to shouldering decisional responsibility; reinforces the need to allow time for deliberation, especially with other stakeholders; and reassures clinicians that consensus, albeit welcome, need not be the goal of shared decision-making.

**Conclusions and Relevance:**

This primer portrays a reflective clinician who is aware of power asymmetry, patient vulnerability, risk communication, health literacy, agenda setting, and goal clarification. It envisages a clinician who is curious about personal perspectives and who can offer collaborative, iterative, and deliberative steps.

## INTRODUCTION

Fifty years after Veatch first described the idea of shared decision-making,^1^ we lack a short explanatory introduction, a primer, that considers the complexity of using the approach in clinical practice. Numerous definitions and descriptions have been published, often led by social scientists^2–4^. Still, definitions and descriptions lack attention to the workflow required of clinicians, especially when interacting with people with limited resources, low expectations, or meager agency experiences. For example, a recent definition is based on the assumption that two, and only two, agents with full autonomy arrive at a mutually agreed decision^5^. We disagree: these idealistic assumptions do not match the conditions observed in healthcare interactions. Achieving shared decision-making needs a different approach when supporting a 35-year-old researcher with breast cancer striving to preserve her fertility versus a 77-year-old pensioner with limited literacy and numeracy facing a choice between surgery and radiotherapy for lung cancer. The researcher typically expects information and autonomy. The pensioner might prefer guidance and decline decisional responsibility. To be helpful, clinicians need a way to think about shared decision-making that helps them navigate the unpredictable yet inevitable range of personalities, contexts, and preferences. In addition, there is often uncertain or limited evidence coupled with the relentless time pressure of clinical schedules. Clinicians, having no objection to the ethical principles of collaboration and deliberation, need practical ways to navigate the challenges of adapting to the diverse needs of people served in the clinic.

Recent decades have brought significant attention to shared decision-making. The approach has been supported in health policy, endorsed by professional associations, and in some countries mandated by legislation.^6^ Healthcare communication skills and evidence-based practice courses have included shared decision-making. Many patient decision aids have been developed to facilitate the approach.^7^ However, shared decision-making, as laid out in key principles,^2^ is not commonly observed in healthcare settings ^8^, and implementation is difficult, especially when there are high stakes and complex information.

We highlight two key barriers to shared decision-making that clinicians have repeatedly expressed.^8^ First, shared decision-making rests on the ethical principle of respecting autonomy. However, people seeking healthcare are often in vulnerable emotional states and, therefore, often far from being confidently autonomous ^9^. Patients and clinicians may be at odds about the goals: a professional aspiration to actively confer agency may be met by patients who wish to decline decisional responsibility, preferring to seek guidance, at least initially. Second, shared decision-making is particularly difficult in encounters where profound differences in power, knowledge, and lived experience exist, often manifesting as a strong deference to clinicians and experts.^10^ Such asymmetry explains some people’s preference to decline a role in decision-making and request guidance.^11^ Conversely, clinicians who assume control exclude others who want a greater decision-making role.

Shifting levels of agency and a preference for different roles influence these interactions. Clinicians will want to also respect people’s autonomy when they decline decision-making roles. Conversely, when people experience positive outcomes from collaborative processes, they may put a higher value on the approach and be less hesitant.^12^ Developing a description of shared decision-making that emphasizes the need for an empathic, flexible, context-, and time-sensitive approach to the challenge of conferring agency may have less risk of undermining patients’ trust and be more readily embraced by clinicians.

Therefore, clinicians would benefit from a statement, a so-called primer, that describes in practical terms the concept and the sequence of tasks that facilitate shared decision-making. We avoided the complex issue of “when” might it be appropriate to deploy shared decision-making.^13^ We know that clinicians interpret the idea of shared decision-making in different ways.^14,15^ Some are strong advocates. Some, however, mistakenly think that the term indicates that the clinician should *share* their professional view about the best way forward or that it is about giving information to fulfill informed consent.^16^ Others, aware of the need to outline and compare alternatives, dismiss shared decision-making as too idealistic because, in their view, giving comparative information and autonomy is unrealistic given time constraints ^17^. Some worry that patients will feel lost, choose the “wrong” option, or regret it later. Multiple interpretations lead to debates about exceptions and concerns about “wrong” choices that are at odds with clinical guidelines.

Our aim was to generate a primer that describes shared decision-making from clinicians’ perspectives and the work required to address the most common obstacles, emphasizing other components. To do that, we invited members of the International Shared Decision-Making Society and, subsequently, a wider group of clinicians and patient representatives to co-produce the proposed statement.

## METHOD

**Figure 1:**
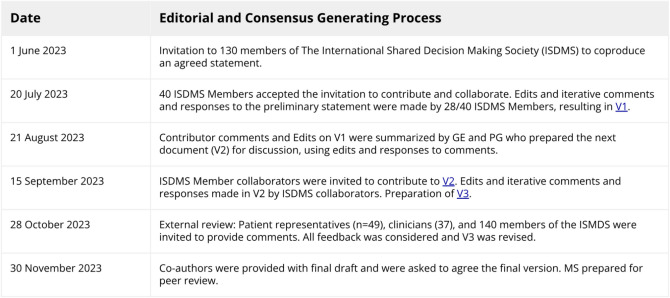
Development of shared decision-making primer statement.

**Figure 2:**
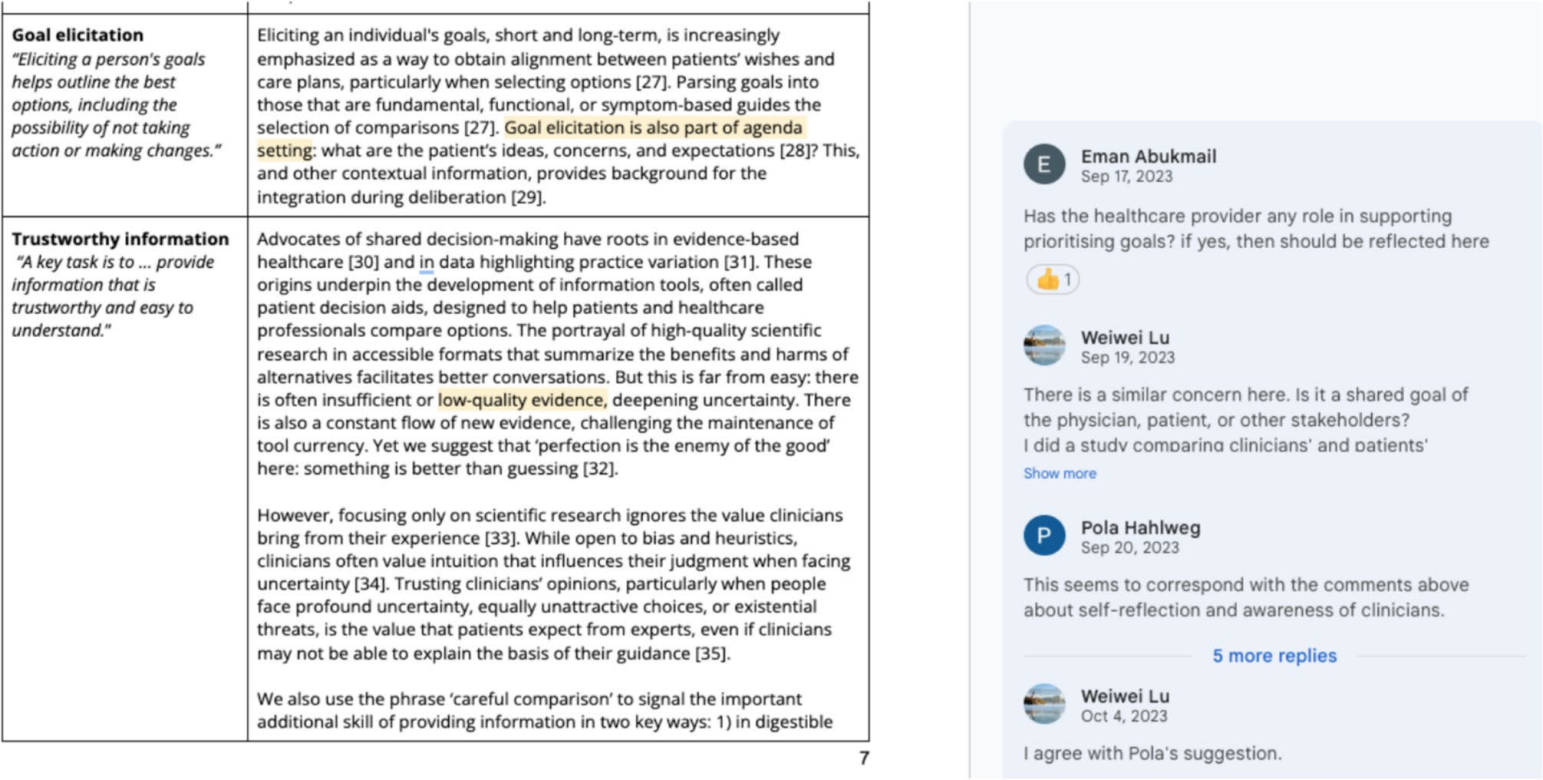
Screenshot: document segment illustrating the editorial process (July to November 2023).

In June 2023, we invited 130 members of the International Shared Decision-Making Society to collaborate on our goal, see Fig. 1 for details, and web links to sample documents. Those (*n* = 40) expressing interest were asked to contribute to an editable cloud-based document (20 July 2023) composed of a preliminary statement describing shared decision-making drafted by GE and PG and to refine, add to, or contest brief summaries of existing definitions. Participants were also asked to propose questions to which others could respond. A selection of those questions is shown in the Box. On 21 August 2023, GE and PG summarized the responses of 28 contributors who had used the comment and reply functions. Based on this archived document, the preliminary statement was substantially revised and resulted in a second document, in which we added a section called “Clarification of Elements.” Contributors were invited to directly edit and comment on this second online draft on 15 September 2023. After a month of asynchronous collaboration that included further edits and the insertion of comments and replies, the second document was closed for further editorial work on 16 October 2023. We monitored the editorial contributions using a spreadsheet and offered those contributors who had made significant input to edits and discussions on both drafts authorship; others were offered acknowledgments. On 28 October 2023, we invited comments on a near-final draft from the following people: (1) 49 patient representatives who had each been asked to be co-authors on separate chapters in a forthcoming 4th edition of the Oxford University Press Textbook of Shared Decision Making; (2) 37 clinician-lead authors of discipline-focused chapters in the same book who had been invited based on their contributions to existing peer-reviewed literature about SDM; (3) all existing members of the ISDM Society as of October 2023 (*n* = 140). Changes were made by GE and PG as a result of comments made by this purposive sample. All authors were asked to review and agree to the final version. These methods are illustrated in Figs. 1 and 2.

**Box **Questions discussed in the initial collaborative document

**Table:** 

• What if an individual declines the offer to engage in decisions? • What situations make it difficult to use shared decision-making? • What if the patient lacks insight into/acceptance of their health condition? • What if the patient has a new diagnosis and has not formed preferences because they lack understanding? • What if the parties disagree about what are reasonable options? • What if there is a lack of, or poor quality, evidence to inform some or all of the options?	

## RESULTS

After three iterative cycles of revisions, we propose a primer for clinicians (see Tables 1 and 2). Twenty-five people from 13 countries contributed as co-authors to the final document; 9 had medical qualifications, 4 had nursing qualifications, and 12 others had a range of backgrounds: for details, see Table 3. A further 30 people in clinical and other roles responded to our invitation to comment (see list in “Acknowledgments”). In the primer, we offer a communication sequence that starts with an invitation and culminates in determination. However, there is *zero intention to prescribe a rigid process:* for maximum benefit, clinicians will adapt to individual contexts and ongoing conversations.

**Table 1:** Shared Decision-Making: a Description

Definition: Shared decision-making in healthcare is a collaboration between individuals that blends science, clinical experience, and people’s preferences when comparing options or plans to determine decisions An explicit invitation: Clinicians usually need to invite and support people to become aware of key decisions to be made, that options exist, and to be encouraged to take part in the decision-making process Non-abandonment: People should also understand that they do not have to take on the burden of decisions and will not be left (abandoned) to make difficult decisions on their own Many people, iterative process: Relatives, friends, and multiple clinicians may be involved, and the process often occurs over more than one conversation Goal elicitation: Eliciting a person’s goals helps outline the best options, including the possibility of not taking action or making changes Trustworthy information: A key task is to help people become aware of the possible range of options or actions and provide information to compare them carefully; information that is trustworthy, balanced, and easy to understand. Being open about uncertainty or the lack of good information is equally vital Deliberation: perspective elicitation and determination: Arriving at, or deferring, a decision involves dialogue: listening and eliciting questions, views, emotions, fears, priorities, and preferences of the people involved. The aim is to co-produce, if possible, a preference for the option or plan that is considered best at the time while leaving it open to review

**Table 2:** Shared Decision-Making: Expanded Descriptions

Descriptive element	Expanded descriptions
**An explicit invitation** “People usually need to be invited and supported by clinicians to be aware that options exist, and are encouraged to take part in the process of deciding.”	Who offers the invitation and when will vary, and it would be best if care teams were also aware and supportive. If the patient (and/or family) is not explicitly invited, there is a significant risk that people will not realize that a choice needs to be made and that they could have a role in deciding There may also be a preparation phase: an orientation to the condition and what may be possible^18^ Collaborating with a clinician about decisions is new to many and might be counter-cultural for some. People will hesitate and have concerns. Without a careful explanation of motives, trust might be at risk: people may consider that such a clinician lacks knowledge, confidence, or both. They may think the clinician is absolving responsibility for recommending action or treatment, unwilling in patients’ eyes to undertake their expert role^19^ It is, therefore, best to explicitly say that the offer to collaborate is to ensure that decisions are informed by patients’ perspectives: that a wise plan requires understanding how people think and feel. Patients should understand that a health professional is willing, if asked, to make recommendations but will do so only after becoming aware of personal perspectives and contexts. As in all decisions, the process will combine rational and emotional elements. Clinicians should also adopt this approach and impress on people that they will work as a team to make decisions,^20^ even if the choices are between a rock and a hard place. As our patient representatives said, shared decision-making is more than asking at the end of a visit: “Is this OK for you?”
**Non-abandonment** “People should understand that they do not have to take on the burden of decisions and that they will not be left (abandoned) to make difficult decisions on their own.”	People might fear being coerced into making a decision or being abandoned to face a difficult decision alone.^21^ Non-abandonment means reassuring patients that they do not have to shoulder decisional responsibility alone and will be supported no matter how long or complex the illness. The role patients wish to play may not be clear at first and may evolve. Even if a person prefers to be guided, collaboration can continue, provided a clinician understands context, goals, and priorities
**Many people** “Relatives, friends, and multiple clinicians may be involved …”	The process cannot be assumed to be a dyadic interaction, even if initiated by one healthcare professional. Other healthcare professionals will be part of the process at different levels of input and expertise; the potential combinations are vast.^22^ In this way, clinical teams also need to support this approach explicitly. Interdisciplinary teams often become actors in decisions, and the integration of patient preferences in clinical team discussions is often neglected^23^ The patient may also seek the advice of people with similar expertise or interact with several professionals with different skills and knowledge. Decision-making differs across cultures.^24^ Relatives and others will be involved, to varying extents, often supportive, but not always ^25^. It becomes clear that deliberations about specific decisions or future actions will be distributed across various actors^26^ Special consideration is required when decisions involve children or young people^27^ or where cognitive capacity is limited or absent.^28^ The challenge at any given healthcare interaction is to assimilate contributions, assess the maturity of the deliberation, and, where possible, support a determination that can be confidently supported
**Iterative process** “… and the process often occurs over more than one conversation.”	Acknowledging that shared decision-making may be distributed across many interactions also means accepting that the process may be distributed across time, even in a dyadic situation. Typically, the more complex the decision or problem, the more time is required. Supporting people to develop informed and well-considered preferences is often an extended process with system implications, demanding meticulous record-keeping and scheduling that prioritizes continuity. However, these are not directly related to the interpersonal communication skills that are the focus here
**The range of options** “A key task is to help people become aware of the possible range of options or actions and provide information to compare them carefully …”	Awareness that a choice of options exists is the cornerstone of shared decision-making.^29^ Options cannot be considered if they are left unspecified. The option of taking no action, sometimes called watchful waiting, declining a test or treatment, or perhaps de-escalating an existing treatment, is often left unmentioned and sometimes viewed negatively by clinicians and patients. In some situations, it may be necessary to convey “no action” or postponement as valid strategies that will not lead to a withdrawal of care but rather be respected Clinicians’ willingness to offer options or explain various actions varies and will strongly influence whether and how collaboration occurs. Genuine clinical equipoise is rare, yet decisions or plans are always sensitive to patient preferences. Therefore, deciding which options to offer and describe is critical and complex. As medicine advances, the range of options expands. Increasingly, economic and policy issues will determine availability. Health systems seldom offer a full range of options: legislation, local or insurance policies, and guidelines will limit options Many factors guide how a clinician portrays options, including knowledge, emotions, biases, and habits. Patients will also become aware of options, especially if exposed to advertising. Our brief definition of shared decision-making will not address how best to arrive at a reasonable range of options
**Goal elicitation** “Eliciting a person’s goals helps outline the best options, including the possibility of not taking action or making changes.”	When selecting options, eliciting individual goals, short and long-term, may help achieve alignment on what is possible. Parsing goals into those that are symptom-based, functional, or fundamental could guide the selection of comparisons.^30^ Goal elicitation is also part of agenda setting: what are the patient’s ideas, concerns, and expectations?^31^ This, and other contextual information, provides background for the integration during a deliberation step^32^
**Trustworthy information** “A key task is to … provide information that is trustworthy, balanced, and easy to understand. Being open about uncertainty, and oftentimes the lack of good information is equally vital.”	Shared decision-making brings together parallel developments in evidence-based medicine and patient-centered care.^33^ These streams underpinned the development of tools, often called patient decision aids, designed to help patients and healthcare professionals compare options ^7^. Summaries of high-quality scientific research pointing to the benefits and harms of alternatives in balanced, accessible formats facilitate better conversations, especially where there is uncertainty. But this is far from easy: information about the burden of treatments is often missing, and data about selected outcomes is given priority at the expense of outcomes that could have more salience to patients.^34^ There is also often insufficient or low-quality evidence, deepening uncertainty: a limitation clinicians must also share.^35^ The constant arrival of new research makes it difficult to maintain tool currency. Yet we suggest that “perfection is the enemy of the good” here: something is better than guessing^36^ However, focusing only on scientific research ignores the value clinicians bring from their experience.^37^ While open to bias, clinicians often value intuition that influences their judgment when facing uncertainty.^38^ Trusting clinicians’ opinions, particularly when people face profound uncertainty, equally unattractive choices, or existential threats, is the value that patients expect from experts, even if clinicians may not be able to explain the basis of their guidance^39^ We also use the phrase “careful comparison” to signal the important additional skill of providing information in two key ways: (1) in digestible bites rather than overwhelming volume, and (2) in formats that make comprehension easier. Communicating comparative data and risk probabilities is a difficult task: a competent practitioner will understand the influence of risk formats, framing, and the beneficial use of images and other visual formats^40,41^
**Deliberation: Perspective elicitation** “Arriving at, or deferring, a decision involves dialogue: listening, eliciting questions, views, emotions, fears, priorities, and preferences of the people involved.”	When goals and options are outlined and contextual information understood, the next two collaborative phases of shared decision-making can proceed. Personal perspective elicitation is about checking views, emotions, concerns, priorities, and preferences related to the decision.^42^ An individual’s values, beliefs, and experience with a condition will be influential. Moreover, some people will need support and time to think and talk to others before they share their opinions. Practitioners may find it helpful to park their recommendations until patients have expressed their thoughts. Being able to pause, listen, facilitate trust, and create a psychologically safe space for patients are key clinical skills
**Deliberation: Determination** “The aim is to coproduce, if possible, a preference for the option, at that time, that is considered best, while left open to review.”	The next deliberation step is to determine which option is considered best.^43^ While some determinations are irreversible, such as undergoing surgery, others are less so, as in the constant decisions involved in living with a long-term illness, so offering to revisit and review decisions will be highly valued It is not uncommon to experience deferment as an intermediate determination: choosing not to make a decision is itself a decision, and often one that initially may feel more comfortable.^44^ Clinicians, when faced with patients who prefer to delay or defer, often have to address their preference for action. Offering a plan to review may help. Clinicians will also need to address their comfort levels when patients arrive at decisions that are not aligned with clinical guidelines or with their views.^45^ Agreement may occur, but this is not the goal of shared decision-making. More important is that people experience a compassionate process that builds trust and that can be revisited if needed

**Table 3:** Author Discipline, Institution, Location, and Contribution

Name	Discipline, content expertise	Institution	Geographical location	A description of the member’s contribution
Eman Abukmail	Clinical tutor and doctoral student (evidence-based medicine and shared decision-making), former general practitioner	Institute for Evidence-Based Healthcare, Bond University	Queensland, Australia	Coauthor: provided comments on first and second drafts
Marla Clayman	Researcher	Center for Health Organization and Implementation Research, Veterans Health Administration	Chicago, USA	Coauthor: provided comments on first and second drafts
Adrian Edwards	Family doctor, researcher, and educator	Division of Population Medicine, Cardiff University	Cardiff, UK	Coauthor: provided comments on first and second drafts
Glyn Elwyn	Researcher, former primary care clinician	The Dartmouth Institute for Health Policy & Clinical Practice	VT, USA	Co-lead author: initiated, created, and edited drafts
Jeanette Finderup	Clinical nurse specialist (kidney disease), associate professor in nephrology and patient involvement	Aarhus University Hospital & Aarhus University	Aarhus, Denmark	Coauthor: provided comments on first and second drafts
Alana Fisher	Researcher, consumer engagement manager	The eCentreClinic, School of Psychological Sciences, Macquarie University, Australia MindSpot, MQ Health, Macquarie University, Australia	Sydney, Australia	Coauthor: provided comments on first and second drafts
Stuart W. Grande	Medical sociologist and community-based researcher	University of Minnesota, School of Public Health, Division of Health Policy and Management	Minneapolis, USA	Coauthor: provided comments on first and second drafts
Pål Gulbrandsen	Researcher, former primary care clinician	Institute of Clinical Medicine, University of Oslo	Oslo, Norway	Co-lead author: initiated, created, and edited drafts
Pola Hahlweg	Researcher, clinical psychologist, psycho oncologist, clinical ethics consultant; Fellow in Biomedical Ethics	University Medical Center Hamburg-Eppendorf, Germany; Harvard Medical School, Boston, MA, USA	Hamburg, Germany	Coauthor: provided comments on first and second drafts
Tammy Hoffmann	Researcher, clinical epidemiologist, occupational therapist	Institute for Evidence-Based Healthcare, Faculty of Health Sciences and Medicine, Bond University	Gold Coast, Australia	Coauthor: provided comments on first and second drafts
Wen-Hsuan Hou	Professor, attending physician	Department of Physical Medicine and Rehabilitation, Taipei Medical	Taipei, Taiwan	Coauthor: provided comments on first and second drafts
María José Hernández-Leal	Nurse, primary care, health economics, healthcare management	School of Nursing, University of Navarra, Spain. Millenium Nucleus on Sociomedicine, Chile	Navarra, Spain	Coauthor: provided comments on first and second drafts
Hannah Leavitt	Research assistant	The Dartmouth Institute for Health Policy & Clinical Practice	TX, USA	Coauthor: provided comments on first and second drafts
Debra Leung	Staff specialist anesthesiologist, researcher, PhD candidate	Peter MacCallum Cancer Centre	Melbourne, Australia	Coauthor: provided comments on first and second drafts
Weiwei Lu	PhD candidate in social and behavioral science	Department of Social and Behavioural Sciences, City University of Hong Kong	Hong Kong, China	Coauthor: provided comments on first and second drafts
Lars Mandelkow	Researcher, educator, family therapist	Ansgar University College (Kristiansand) & Center for Shared Decision Making, University Hospital of North Norway (Tromsø)	Kristiansand and Tromsø, Norway	Coauthor: provided comments on first and second drafts
Kristen Pecanac	Researcher, former critical care nurse	University of Wisconsin-Madison, School of Nursing	Madison, USA	Coauthor: provided comments on first and second drafts
Arwen H. Pieterse	Researcher in medical decision-making and communication, educator, cognitive psychologist	Leiden University Medical Center	Leiden, The Netherlands	Coauthor: discussions with lead editors and comments on the second draft
Amy Price	Researcher, editor methodologist, patient author	The BMJ, The Dartmouth Institute for Health Policy & Clinical Practice	Florida USA	Coauthor: discussions with lead editors and comments on the second and third draft
Jannicke Rabben	Cancer nurse, PhD candidate; shared decision-making in palliative cancer care	University of Agder, Faculty of Health and Sport Sciences, Department of Health and Nursing Science	Kristiansand, Norway	Coauthor; provided comments on the first and second drafts
Paula Riganti	Family doctor, educator, and researcher	Hospital Italiano of Buenos Aires, Argentina, University of British Columbia, Canada	Vancouver, Canada	Coauthor: provided comments on first and second drafts
Michael Sanatani	Medical oncologist (lung and GI), educator, and researcher	Schulich School of Medicine & Dentistry, Western University	London, Ontario, Canada	Coauthor: provided comments on first and second drafts
Fülöp Scheibler	Medical sociologist, systematic reviewer	National Competency Center for Shared Decision Making, University Schleswig–Holstein	Cologne, Germany	Coauthor: provided comments on first and second drafts
Elise Schoefs	PhD candidate and researcher in pharmaceutical sciences	KU Leuven Department of Pharmaceutical and Pharmacological Sciences	Leuven, Belgium	Coauthor: provided comments on first and second drafts
Owen A. Taylor	PhD student in public health and primary care	Cardiovascular Epidemiology Unit, Department of Public Health and Primary Care, University of Cambridge	Cambridge, UK	Coauthor: provided comments on first and second drafts
Kathrene D. Valentine	Researcher, methodologist	Massachusetts General Hospital, Department of General Internal Medicine	MA, USA	Coauthor: provided comments on first and second drafts
Richard Wexler	Retired gastroenterologist and former chief medical officer at the Informed Medical Decisions Foundation	Informed Medical Decisions Foundation	Boston, MA, USA	Coauthor: provided comments on first and second drafts

## DISCUSSION

The primer statement puts the work of explicitly eliciting and integrating personal perspectives when making decisions at the heart of clinical practice, informed by science and clinical experience. The essence is to ensure that there is an effort made to ensure a good fit between a patient’s goals and the choice to be made. There are times when constraints make the task difficult to achieve fully. Yet, many clinicians can get close by making efforts to inform and guide an anxious patient so that decisions take account of personal situations and contexts. This primer, co-produced by this group of clinicians, patients, and others, differs from previous definitions and descriptions because it was generated to address the most frequent problems clinicians report as they strive to accomplish shared decision-making.

Clinicians often say that patients resist being given a role in decisions. This primer emphasizes the importance of a clear invitation and a careful justification when initiating a process that might be novel for many people. They could reinforce that involvement is voluntary, not mandated, and no patient will be abandoned to face tough decisions alone; that creating a sense of unhurried time, or offering further discussion, would facilitate deliberation, especially where family members or other stakeholders need to be involved. This primer reminds clinicians that mutual agreement about next steps, although welcome, is not the primary goal of shared decision-making.

The primer portrays a reflective clinician,^46^ aware of power asymmetry, patient vulnerability, distributed actor involvement, risk communication, health literacy, agenda setting, and goal clarification.^47^ It is a portrait of a curious, capable communicator who offers iterative, collaborative, deliberative steps—the pinnacle of patient-centered care.^48^ These values, attitudes, and skills are not accidentally developed: they are carefully curated.

### Strengths and Weaknesses of the Method

We fostered a more open, emergent, and formative process for developing consensus by encouraging ongoing debates and discussions among contributors over many months, allowing debate, reflection, and ongoing refinement of the document. The iterative approach is documented in multiple archived records (see Fig. 1 for access). We limited authorship to active voluntary contributors, ensuring representation from a significant number of patient representatives, clinicians in active practice, and researchers in the field. Our approach suffers a number of weaknesses. There is a strong selection bias: the authors and other contributors are known advocates of shared decision-making: the debates focused on details versus the principles of respecting agency and autonomy. We did not have authors or commentators representing populations with low literacy or limited education. We had relatively low representation of cultures that place a lower value on individual autonomy. A more formal consensus method, such as a modified Delphi method, would have provided more formal quantitative evidence of agreement. However, despite these flaws, the inclusive, asynchronous, detailed parallel editing we witnessed facilitated significant author engagement over many months across time zones and multiple cultural contexts.

### Results in Context

The term “shared decision-making” has become widely known and debated^8,49^.^13,50–55^ A seminal article in 1997, led by a sociologist, emphasized shared decision-making principles within dyadic encounters,^2^ and led to a surge of interest. Reviews of definitions in 2006 and 2019, despite identifying similar elements,^3,4^ suggested that the lack of consensus about shared decision-making was, in part, responsible for implementation challenges. In 2025, using the title “saving shared decision making,” Opel et al. suggested that the use of the term equipoise, if interpreted to mean perfect balance, has been counter-productive. Equipoise, they argued, was neither “necessary nor sufficient”.^56^ However, there should be no argument with the proposal that SDM is relevant when it is reasonable to offer alternatives, recognizing that perfect evidential balance is rare. It is also clear that SDM has limits,^13^ especially when societal or professional obligations are not aligned with a person’s preference, such as a declining vaccination against measles. The suggestion to use the term “everyday SDM” for situations where clinician recommendations are legitimate,^57^ despite possible contrary individual preferences, muddies the water. The ongoing debates feel like dances on heads of pins, and have led to our goal of developing a clearer description of how SDM can be accomplished, recognizing that while it is true that medical practice has been slow to prioritize patients’ perspectives, advocates of SDM have also overlooked the difficulties clinicians face.

### Implications

This primer details how clinicians can manage tasks such as invitation, non-abandonment, personal perspective elicitation, and collaborative deliberation. Those responsible for health policy and designing the structures and schedules for clinicians should actively motivate these behaviors with a mix of intrinsic and extrinsic rewards. Established clinicians may struggle to prioritize a better understanding of their patients’ perspectives. The in-the-moment cognitive flexibility required to form a different dialogue may take time to master. Shared decision-making is unlikely to take less time, but cultivating this approach could bring more variety and joy to clinical work.

## Declarations

### Patient Representatives

The following patient representatives were asked to comment and edit the article:

E. Chandlee Bryan, MEd, patient representative and a person with multiple sclerosis.

Jan Faldt Bentsen, patient representative, Chair of Patient & Relative Council at Sygehus Lillebaelt, Kolding, Denmark.

Hannah Leavitt, MPH, patient representative with dysautonomia, The Dartmouth Institute for Health Policy & Clinical Practice, New Hampshire USA.

Ellen McEvoy, patient representative, participant in the MAMMI Study, Ireland.

Nadine Montgomery Allam, patient representative, Patron of Birthrights, Medical Lawyer at Levy and McRae solicitors, and Class Tutor for Law of Delict at the University of Strathclyde, Scotland.

Johannah Ruddy, MEd, patient representative, doctoral candidate, Population Health, Campbell University School of Health Sciences, USA.

Henning Søndergaard, patient advocate and psychologist, Danish Kidney Association, Nyreforeningen, Denmark.

Elaine Taylor, patient representative, MSK, Hywel Dda Health Board, Wales.

Janice Tufte, patient representative, involved in Health Systems Research and Quality Improvement Efforts, Seattle, WA, USA.
